# Performance of high-translucent zirconia CAD/CAM fixed dental prostheses using a digital workflow: A clinical study up to 6 years

**DOI:** 10.1016/j.jds.2022.07.023

**Published:** 2022-08-13

**Authors:** Mustafa Baris Guncu, Guliz Aktas, Ilser Turkyilmaz, Joanna Nicolette Gavras

**Affiliations:** a Department of Prosthodontics, School of Dentistry, Hacettepe University, Ankara, Turkey; b Department of Prosthodontics, New York University College of Dentistry, New York, NY, USA

**Keywords:** CAD/CAM, Digital dentistry, Digital impression, Intraoral scans, Prosthesis, Zirconia

## Abstract

**Background/purpose:**

Zirconia has recently become a popular material for fixed restorations. The purpose of this study was to use a digital workflow to fabricate monolithic zirconia fixed dental prostheses (FDPs) and assess the connection between variable connector sizes compared to their clinical performance.

**Materials and methods:**

Clinicians evaluated monolithic zirconia FDPs in 58 patients. After definitive impressions were made, stone casts were obtained. The stone casts were scanned to a standard triangle language (STL) file. A digital wax up was fabricated, and corresponding provisional restorations were milled. Final FDPs were fabricated from a high-translucent zirconia material. During digital fabrication, the connector area of each FDP was recorded while meticulous attention was paid to ensure that the connector cross-sectional area was ≥9 mm^2^ for the 3-unit restorations (pontic to retainer) and ≥12 mm^2^ for the 4-unit restorations (pontic to pontic). Biological an technical outcomes of the FDPs were performed at 1 week, 6 months and then annually for 6 consecutive years.

**Results:**

A total of 23 men and 35 women received a total of 63 full-contour zirconia FDPs in the posterior regions and were observed for a time period ranging between 50 and 70 months. No decementation occurred and no caries were detected during the observation period, however signs of gingivitis were detected in 4 patients. The dimension of the connector areas was 12 mm^2^ in the two broken 4-unit FDPs.

**Conclusion:**

The results of this study suggest that the use of digital scanning and milling to fabricate monolithic zirconia FDPs of posterior regions may be an acceptable alternative restorative approach to traditional metal-ceramic restorations.

## Introduction

Fixed dental prostheses (FDPs) are commonly used restorative procedure to replace missing teeth.^1^, ^2^, ^3^ Recently zirconia has become an increasingly popular material that offers more advantages over their comparative counterparts.^4^, ^5^, ^6^, ^7^, ^8^, ^9^ Traditional materials such as all-ceramic or porcelain-fused-to-metal (PFM) have been used, however, these materials have inherent shortcomings such as fracture and/or chipping leading to failure.^10^, ^11^, ^12^ Additionally, the aesthetic limitations of PFMs could be considered a failure by patient preference standards. Zirconia, especially high translucent ones,^13^, ^14^, ^15^ on the other hand has a greater strength capacity compared to other ceramic materials to bare the occlusal load on masticatory force and is significantly more aesthetic than PFMs. This allows the restoration to perform and appear closer to natural dentition allowing patients a greater level of satisfaction with the procedural outcomes.^13^^,^^14^

While monolithic zirconia does improve on many qualities of traditional materials, it still does have its own fundamental limitations.^10^, ^11^, ^12^ Fracture due to flexural stress under occlusal and rotational forces do still exist and must be studied to determine the success of the restorative material and overall procedure. As the span of the restoration increases, additional directions of force are added into the physics of the restoration. Previous studies have attempted to determine the connector size (pontic-retainer or pontic–pontic) to determine if there is an optimal design necessary for successful FDP fabrication.^16^, ^17^, ^18^ It has previously been suggested that for a 3-unit and 4-unit FDP the connector size of 9 mm^2^ and 12 mm^2^ respectively is considered clinically acceptable.^16^^,^^17^ These numeric values have been indicated for the more traditional restorations, therefore when using a novel material such as zirconia it is imperative to determine if the same values are relevant.

Aside from the new developments in materials, integration of a digital workflow into the clinical setting has greatly improved the quality and speed of treatment provided.^19^, ^20^, ^21^, ^22^, ^23^ Not only do they streamline the fabrication, the accuracy and ease of treatment is vastly increased. The use of computer aided design and computer aided manufacturing (CAD/CAM) technology as well as intraoral scanners (IOS) have revolutionized dental practices allowing for greater long-term success of treatments.^19^^,^^20^

IOS have substantially improved accuracy and clinical performance in treatment outcomes.^22^^,^^24^^,^^25^ Their use allows clinicians to have a digital rendition of the patients’ teeth into a 3-D scanned model. This direct digitization aids in every step of treatment planning, patient communication, as well as actual execution of successful treatment. It allows clinicians to plan interim restorations that patients can approve of without waste of material.^22^ It can also allow a patient to receive a final restoration in a single visit, thus increasing the availability to treating patients, who would be otherwise limited by time and location constraints.

The digital design can be mocked up and checked prior for any issues that would cause a failure of the restoration and need to be sent back to the laboratory for adjustment or refabrication.^19^^,^^22^ This allows for less materials (e.g., ceramic) to be wasted and keeps the cost of these treatments down increasing the accessibility of treatment to patients. Once everything is checked the final milling process can occur, and the restoration can be milled out of a single block of material as opposed to traditional layering techniques as seen with PFM restorations. This will eliminate the failure of separation that may occur between the layers, therefore decreasing the need for a patient to remake their restoration sooner than clinically predicted.

From a clinical research perspective, scanning allows for a more accurate readings giving one the ability to quantify data at the microscopic level. This precise and accurate data gives clinicians the ability to draw more understanding for any objective they may have set out to study. At this point it may be seen that as the span of scanning for an arch, the accuracy does decrease,^26^, ^27^, ^28^ but as further developments are made it may be seen that IOS may entirely replace conventional forms of taking impressions.

Of course, as with all novel concepts, accurate implementation, and use of said workflow is key. If used incorrectly a clinician or patient will not be able to benefit from the techniques provided. There is a learning curve and an initial investment of time and money that is needed in order to implement these tools into everyday practice.^29^^,^^30^

Through scanning, digitization and milling, this study aimed to evaluate the clinical performance of monolithic zirconia fixed dental prostheses with variable connector sizes and determine if there was a connection between the contact area and fracture resistance for the FDPs under different contributing variables.

## Materials and methods

### Study design and data collection

This study was performed in February 2022 and run retrospectively in a dental faculty practice to evaluate monolithic zirconia multiunit FPDs. Authors evaluated the records of 58 patients who had received monolithic zirconia FPDs to replace one or two missing teeth during the time span of January 2016 to December 2017. An approval for this study was obtained from our school's Institutional Review Board (Number: GO 22/285). Prior to treatment, the participants were informed about the other alternative treatments and the advantages and disadvantages of the monolithic zirconia material and allowed to choose if they wished to move forward with this treatment. Each participant signed an informed consent form for this study.

Patient selection was based on the following inclusion criteria: (1) 1 or 2 missing posterior teeth (either premolar, molar, or premolar and molar) requiring replacement by means of FDPs of between 3 and 4 units; (2) abutment teeth presenting stable periodontal health; no signs of active bone resorption, furcation involvement, or periapical pathology; (3) moderate to good oral hygiene and low caries risk; (4) good general health without severe physiologic or psychologic conditions. Excessive parafunctional habits were not considered an exclusion criterion.

The following data were considered: age, sex, number of prosthetic units, location of abutments and pontics, existing condition of abutment teeth (vital or endodontically treated), opposing dentition, zirconia and luting cement trademark, use of an occlusal splint or signs of bruxism, dimensions of connector cross-sectional area of each bridge (abutment to pontic and pontic to pontic), time in mouth from placement to the last examination. Each restoration was assessed by an experienced clinician (GA), who was not involved in patient treatment, in terms of marginal integrity, surface, color, and anatomic form. Evaluation of each category was rated as excellent, acceptable, or unacceptable according to the California Dental Association (CDA) quality evaluation system. All the complications leading to a replacement of the restoration (biological or technical) were defined as a failure.

### Treatment procedures

A consistent team of one skilled clinician (MBG) and a one dental technician provided patient care adhering to strict prosthodontic protocols. Occlusal surface reduction of abutment teeth ranged from 1.5 to 2 mm, and axial reduction ranged from 1 to 1.5 mm, with a convergence angle of 10- to 20-degrees. A 1.4 mm round-end cylinder diamond bur (Komet, Brasseler, Lemgo, Germany) was utilized to create a 0.7 mm chamfer finish line in the preparations. After all line angles were rounded off, gingival displacement was achieved, then definitive impressions were made with polyvinyl siloxane impression material (Elite HD+, Zhermack SpA, Rovigo, Italy). Temporary restorations were fabricated by using a bis-acrylic resin (Acrytemp, Zhermack SpA, Rovigo, Italy) and a matrix, and then cemented with a eugenol-free temporary cement (RelyX™ Temp NE, 3 M ESPE, Neuss, Germany). Impressions of the counter arch were made with the same impression material and interocclusal bite registration records were made (Blu-Mousse, Parkell, New York, NY, USA). The working casts and the abutment dies were fabricated with a Type IV dental stone (Klasse 4 Dental, Augsburg, Germany). Each stone cast was then scanned to an STL file. The file was then uploaded to a programming software that allowed the practitioner to create a digital wax up. Provisional restorations were milled from each corresponding digital wax up and then temporarily cemented.

### Fabrication of the restoration

The workflow of the fabrication of FPDs was as follows; firstly the provisional restorations that were milled from an acrylic material in a laboratory served to check esthetics and function. The anterior guidance and occlusal balance were both adjusted intraorally to establish the simultaneous bilateral contacts of opposing teeth during eccentric movements. After the temporary restorations were sent back to the dental laboratory, the technician rescanned and virtually superimpositioned them onto the previous design by using the same software (Dental Wings Inc., Montreal, Canada). A 80-micron space was added into the design for cement space.

Definitive FDPs were fabricated as full-contour zirconia restorations from a presintered high translucent zirconia material (Katana HTML, Kuraray Noritake Dental Inc., Okayama, Japan). During the designing, each connector's cross-sectional area of the restorations was recorded. Meticulous attention was paid to ensure that connector cross-sectional area was ≥9 mm^2^ for the 3-unit restorations (pontic to retainer) and ≥12 mm^2^ for the 4-unit restorations (pontic to pontic) with a rounded curvature.

The thickness of the restorations was at least 1 mm. The sintering procedure was performed strictly adhering to the manufacturer's instructions. By a single clinician, restorations were evaluated intraorally to ensure accurate seating by using fit checker, and proper occlusal contacts by using articulating paper. If necessary, occlusal adjustments were done with a fine diamond zirconia intraoral polishing rotary instrument under water cooling.

Paste-type surface staining (Cerabien Zr, Kuraray, Okayama, Japan) material was applied for the characterization of full-contour zirconia. FPDs were glazed (Cerabien Zr) according to the manufacturer's recommendation at 850 °C. The bonding surface of the abutment tooth was rinsed with water spray and a conditioner (Fuji Plus Conditioner, GC, Tokyo, Japan) was applied for 20 s. After ultrasonically cleaned in 95% ethyl alcohol for 5 min, restorations were cemented with a resin-modified glass ionomer cement (Fuji Plus).

### Follow-up

After delivery of the FDPs, a skilled clinician (G.A.) without any prior involvement with the experiment was chosen to evaluate the biological and the technical outcomes at 1 week, 6 months and then annually for 6 subsequent years. The esthetics and function of the prostheses were evaluated in terms of marginal integrity, surface, color, and anatomic form according to the California Dental Association (CDA) quality evaluation system as excellent, acceptable, and unacceptable (correction or replacement).^31^ Any complication (biological or technical) that indicated replacement of the prosthesis was classified as a failure.

### Statistical analysis

The data for 63 full-contour zirconia FDPs were statistically analyzed with a software (SPSS version 25, IBM Corp, Armonk, NY, USA). For each FDP, the time to failure and complications were recorded. The survival of the restorations compared to subcategories of number of units, bruxism, jaw, antagonist and pontic location were displayed using Kaplan–Meier survival curves. The significance of differences between survival curves was determined with the log-rank test. A *P*-value < 0.05 was considered statistically significant.

## Results

Fifty-eight patients (23 men, 35 women) between the ages of 24 and 72 (mean age 47 ± 11.7) received 63 full-contour zirconia posterior FDPs (Table 1). The observation period of this study ranged from 50 to 70 months upon completion of treatment. The following observations were collected and recorded for further analysis. Of the total 63 FDPs, 44 were 3-units and 19 were 4-units (with two adjacent pontics). While 25 (56.8%) of the 3-unit FDPs were in the mandible and 19 (43.2%) were in the maxilla, 5 (26.3%) of the 4-unit FDPs were in the mandible and 14 (73.7%) were in the maxilla. There were 30 (47.6%) natural teeth and 33 (52.4%) fixed restorations (implant- or tooth-supported) in the opposing dental arch. Signs of bruxism, or the use of an occlusal splint for the duration of this study was noted for 27 (45%) of the participants. In 45 of the 63 total FDPs, both abutment teeth were vital. In the remaining 18 FDPs either one or both abutment teeth have been endodontically treated. No decementation has occurred on any of the restorations. No caries were detected, however, signs of gingivitis were noted in 4 patient.

**Table 1:** Detailed information about the patients, dentitions and fixed dental prostheses (FDPs).

		Number	Survival rate %	
Bruxism	(+)	27	88.9	***P* < 0.05**
	(−)	36	100	
Gender	Female	40	92.5	*P* > 0.05
	Male	23	100	
Jaw	Maxilla	35	94.3	*P* > 0.05
	Mandible	28	96.4	
Opposite Dentition	Natural teeth	30	90	***P* < 0.05**
	Fixed Restoration	33	100	
Pontic	Premolar	14	100	*P* > 0.05
	Molar	30	96.7	
	Premolar + Molar	19	89.5	
Unit	3-units	44	97.7	*P* > 0.05
	4-units	19	89.5	

The CDA quality evaluation system was used to evaluated the end result of treatment. It was found that (except for three bridges that were replaced during the follow-up period) the CDA scores of the remaining ones were as follows; 52 (87%) were rated as excellent and 8 (13%) acceptable for marginal integrity. Anatomic form was noted as 39 (65%) excellent, 21 (35%) acceptable. Color and surface texture was rated as 17 (28%) excellent, 43 (72%) acceptable. Survival rate of 3- and 4- unit FDPs were 97.7% and 89.5% respectively, which indicated no statistical difference (*P* > 0.05) (Table 1). After a 60.1 ± 13.2-month follow-up period, the overall survival rate was recorded as 95.2%.

Two of the 4-unit bridges were broken at the pontic–pontic connector area. It was noted that on both broken bridges the dimension of connector area was 12 mm^2^ (Table 2). The distribution of the abutments and pontics and the dimensions of cross-sectional area of the connectors are presented in. One prosthesis of 3-unit bridges had to be replaced because of a technical complication; a perforation occurred in the occlusal surface of retainer second lower molar.

**Table 2:** Connector areas of successful and failed fixed dental prostheses (FDPs).

	Mesial connector area	Pontic–pontic area	Distal connector area
**44 three-unit FDPs**	15.41 ± 2.34 mm^2^		16.05 ± 2.55 mm^2^
**19 four-unit FDPs**	15.84 ± 2.46 mm^2^	16.68 ± 2.83 mm^2^	17.11 ± 2.49 mm^2^
**2 four-unit FDPs (broken)**	12.50 ± 0.71 mm^2^	12.00 ± 0.00 mm^2^	13.00 ± 0.00 mm^2^

## Discussion

Previously performed studies have attempted to assess the validity of monolithic zirconia FDPs. Many have demonstrated that monolithic zirconia as the sole restorative material result in successful treatment. Although monolithic zirconia FDPs in the posterior region has already been deemed a viable alternative treatment option, additional information must be gathered as the us of monolithic zirconia is becoming increasingly popular and may possibly replace previously used alternative restorative materials.

Although direct comparisons between the present study and previous studies can not be made as they used different materials and workflows, following studies can be mentioned here. A study by Pelaez et al.,^4^ assessed the clinical performance of 3-unit zirconia posterior FDPs over three years. Authors placed twenty 3-unit FDPs in a total of 17 participants where nine were placed in the mandible and 11 were placed in the maxilla. The Lava system was used to prepare all abutment teeth as well as framework. Final restorations were delivered with a resin cement and were evaluated by two examiners at 1 week, and then each year for 3 consecutive years using the CDA quality evaluation system as done in the current preset study. Gingival, plaque and marginal indices were all evaluated as well as probing depths for the abutment teeth and the contralateral teeth of the restorations. No fractures of framework were observed in any FDP. One restoration was lost due to a biologic complication at the 3-year evaluation. There were no significant variations in the periodontal parameters of either group for the exception of the marginal index. Findings of this study suggest that zirconia 3-unit FDPs in the posterior region are a reliable treatment.^4^ This gives support to the idea that zirconia is a sufficient alterative restorative material that provides just as much strength to traditional metal framework.

Another study by Pontevedra et al.,^32^ demonstrated that a digital workflow and monolithic zirconia was a viable alternative to the commonly used veneered zirconia. Their study design was similar to this present study. They included 60 patients to receive a 3-unit posterior FDP. Similarly to this study's workflow, IOS were used for digital impressions, digital software was used to prepare a design, milling machine was used for fabrication, and final restorations were delivered with resin cement. They observed that the survival rates the monolithic and veneered zirconia were 90% and 100% respectively. Biologic complications were the only reason which three of the monolithic FDPs failed. No framework fracture for either group was observed however with the veneered group the only complication reported was fracture of the veneered ceramic in 4 of the FDPs. These findings suggested that in the posterior region of the oral cavity, monolithic zirconia 3-unit FDPs are sufficient restorations, and may be a better alternative to veneered zirconia FDPs.

While these studies have suggested that the use of zirconia is an appropriate material for the fabrication of an FDP, additional considerations are needed to determine if this restorative method is case specific or can be generalized as the new gold standard of FDP restorations. Other parameters such as increasing span of restoration, connector size design, as well as overall success of restorations in patients who display parafunctional habits need to be considered in order to generalize the claim the monolithic zirconia is a satisfactory restorative material alternative.

The connector areas of these FDPs are a factor that may be considered for design flaws. Previous studies have claimed clinical acceptable values in connector size for the posterior regions of 3-unit FDPs to be no less than 9 mm^2^.^16^^,^^17^ However, in the study performed by Wolfart et al.,^33^ where the connector size was larger, two (8%) FDPs fractured, possibly indicating that the previously clinically acceptable value for a 3-unit FDP is starting at too low of a value. In the case of this study, all 3-unit FDPs have connectors of 15 mm^2^ and 16 mm^2^. Not one of these FDPs fractured, indicating that the clinically accepted value of 9 mm^2^ may be better suited to start at a value closer to those reported in this study. The two 4-unit FDPs that did happen to fracture were in patients who displayed parafunctional habits of bruxism, and the connector size were around 12 mm^2^ and 13 mm^2^ This would indicate how much the connector size influences the success of an FDP and it must be considered in order to guarantee the longevity of the restoration.

A statistical significance was noted in this study for patients who experienced bruxism. In the case of patients with parafunctional habits, out of 27, two of the 4-unit FDPs did fracture resulting in a statistically significant survival rate of 88.9%. It is also noteworthy to reiterate that the connector size of those 4-unit FDPs for the mesial, pontic to pontic, and distal connector area were as follows: 12.50 ± 0.71 mm^2^, 12.00 ± 0.00 mm^2^, and finally, 13.00 ± 0.00 mm^2^ (Table 2). This provides direct support to the idea that the previously clinically accepted value for 4-unit FDPs of 12 mm^2^ in the posterior region may be too low. It is also possible to claim in cases that restorations are fabricated for patients with bruxism, the minimum connector size for posterior regions needs to be greater than 12 mm^2^. Instances where excessive or even general occlusal loads cause compressive stress directly to the connectors of the FDP causing microcracks and ultimately leading to fracture of the FDP was also found in the study performed by Hamza et al.^16^ The idea that a larger connector size may decrease the instance of FDP fracture was substantiated in both this present and previously mentioned study. A larger sample sizes of patients with variable parafunctional habits may be considered in future studies to strengthen the validity of increasing the minimum connector area of FDPs in posterior regions.

In certain cases where patients have parafunctional habits or have different restorations on opposing dentition of the FDP, what was once established as general clinical acceptance may not directly apply to everyday practice. As new considerations are put into practice, the continual successful use of this material with a digital workflow of intraoral scanners and milling machines may lead to conclusive evidence supporting monolithic zirconia FDPs as an acceptable alternative restorative material to the traditional all metal-ceramic and veneered-zirconia.

## Declaration of competing interest

The authors have no conflicts of interest relevant to this article.
